# Evaluating the Accuracy of Artificial Intelligence Models for Early Lung Cancer Detection: Evidence From a Systematic Review

**DOI:** 10.7759/cureus.107774

**Published:** 2026-04-26

**Authors:** Eiman Elzein Abdelrahman Elsheikh, Mohammed Alfatih Mohammed Ramadan, Fatima Ismail Abdelhalim, Esra Bushra Hassan Babiker

**Affiliations:** 1 Acute Medicine, Ashford and St Peter’s Hospitals NHS Foundation Trust, Surrey, GBR; 2 General Medicine, Bedfordshire Hospitals NHS Foundation Trust, Bedfordshire, GBR; 3 Pulmonology, Dr. Mohammad Alfagih Hospital, Riyadh, SAU; 4 Internal Medicine, Muhayil General Hospital, Aseer, SAU

**Keywords:** artificial intelligence, deep learning, diagnostic accuracy, early detection, lung cancer, systematic review

## Abstract

Lung cancer remains the leading cause of cancer-related mortality worldwide, with early detection critical for improving outcomes. While low-dose computed tomography (CT) screening has demonstrated mortality benefits, its implementation is constrained by high false-positive rates and inter-observer variability. Artificial intelligence (AI) has emerged as a promising tool to enhance diagnostic accuracy. This systematic review evaluates the accuracy of AI models for early lung cancer detection. A systematic search was conducted across PubMed, Scopus, Embase, ACM Digital Library, and IEEE Xplore for studies published between January 2021 and December 2025 following the Preferred Reporting Items for Systematic Reviews and Meta-Analyses (PRISMA) guidelines. Studies evaluating AI models for early lung cancer detection using imaging modalities were included. Methodological quality was assessed using the QUADAS-2 (Quality Assessment of Diagnostic Accuracy Studies-2) tool. Ten studies met the inclusion criteria. AI models, predominantly convolutional neural networks, demonstrated high diagnostic accuracy across CT, chest X-ray, and histopathology imaging. Accuracy ranged from 75.2% to 99.17%, with sensitivities between 80% and 100% and specificities from 58.7% to 98.03%. Several studies reported AI performance comparable to or exceeding that of radiologists. Ensemble and hybrid models consistently outperformed single-architecture approaches. Quality assessment revealed moderate to high methodological quality overall. AI models achieve high diagnostic accuracy for early lung cancer detection, with performance often comparable to that of radiologists. AI is best positioned as a complementary tool to augment human expertise. Future research should prioritize prospective designs, external validation, and standardized reporting.

## Introduction and background

Lung cancer continues to represent the foremost cause of cancer-related mortality globally, largely attributable to delayed diagnosis and the asymptomatic nature of early-stage disease [1]. While advances in therapeutic interventions have modestly improved outcomes, survival remains strongly stage-dependent, with early detection offering a markedly better prognosis [2]. The introduction of low-dose computed tomography (LDCT) screening has significantly reduced mortality among high-risk populations; however, its clinical implementation is constrained by high false-positive rates, overdiagnosis, and substantial inter-observer variability among radiologists [3]. These limitations not only increase healthcare burden but also highlight the pressing need for more precise, reproducible, and scalable diagnostic strategies.

Artificial intelligence (AI), particularly deep learning approaches such as convolutional neural networks (a type of AI model designed to recognize patterns in images), has rapidly emerged as a transformative tool in thoracic imaging [4]. By leveraging large-scale imaging datasets, AI models can autonomously learn complex radiographic features associated with pulmonary nodules and malignancy risk, often achieving performance metrics comparable to or exceeding those of human experts in controlled settings [5]. Recent studies have demonstrated promising diagnostic accuracy, with high sensitivity, specificity, and area under the receiver operating characteristic curve (AUROC - a measure of how well a model distinguishes between cancerous and non-cancerous cases), as well as the potential to augment radiologist performance through AI-assisted workflows [6]. Nevertheless, these findings are accompanied by notable heterogeneity in model development, validation techniques, dataset composition, and clinical applicability, raising important concerns regarding reproducibility, external validity, and real-world integration [7].

In light of the accelerating expansion of AI-driven research in lung cancer diagnostics, a rigorous and focused synthesis of current evidence is warranted. Existing reviews have frequently been limited by narrow scope, methodological variability, or insufficient emphasis on standardized diagnostic performance metrics. Therefore, this systematic review aims to critically evaluate the accuracy of AI models for early lung cancer detection by systematically analyzing key indicators such as sensitivity, specificity, accuracy, and AUROC, alongside comparative assessments with radiologist performance where available. Through this approach, the present study seeks to clarify the clinical utility, strengths, and limitations of AI-based diagnostic systems and to inform future research directions and translational implementation in oncologic imaging.

## Review

Methodology

Design and Reporting Standards

This systematic review was conducted in accordance with the Preferred Reporting Items for Systematic Reviews and Meta-Analyses (PRISMA) guidelines [8] to ensure methodological rigor, transparency, and reproducibility. The review process was carefully structured to identify, evaluate, and synthesize the most relevant and recent evidence regarding the diagnostic accuracy of AI models for early lung cancer detection. All stages of the review, including literature search, study selection, data extraction, and quality assessment, were performed following standardized procedures to minimize bias.

Eligibility Criteria (PICOS Framework)

The eligibility criteria for study inclusion were defined using the PICOS (Population, Intervention, Comparison, Outcomes, and Study Design) framework to ensure clarity and consistency (Table 1) [9].

**Table 1:** Eligibility criteria. PICOS (Population, Intervention, Comparison, Outcomes, and Study Design) framework [9]. CT: computed tomography; LDCT: low-dose computed tomography.

Component	Description
Population (P)	Human subjects undergoing screening or diagnostic evaluation for early lung cancer, including high-risk or asymptomatic populations
Intervention (I)	Artificial intelligence-based models (e.g., machine learning, deep learning, convolutional neural networks) applied to imaging modalities such as CT, LDCT, or chest X-ray
Comparator (C)	Radiologist interpretation, standard diagnostic methods, or reference standards such as histopathology or clinical follow-up
Outcomes (O)	Diagnostic performance metrics, including sensitivity, specificity, accuracy, area under the receiver operating characteristic curve (AUROC), and related measures
Study design (S)	Original research studies, including retrospective and prospective diagnostic accuracy studies

Only studies published in English between January 2021 and December 2025 were included to ensure the incorporation of the most recent advancements in AI technologies. Reviews, editorials, conference abstracts without full text, animal studies, and studies lacking sufficient diagnostic performance data were excluded.

Information Sources and Search Strategy

A comprehensive and systematic literature search was conducted across multiple electronic databases, including PubMed, Scopus, Embase, ACM Digital Library, and IEEE Xplore. These databases were selected to ensure broad coverage of both biomedical and technical literature relevant to AI applications in medical imaging. The search strategy combined controlled vocabulary (e.g., MeSH terms) and free-text keywords related to “lung cancer,” “artificial intelligence,” “deep learning,” “machine learning,” and “early detection.” Boolean operators (AND, OR) were used to refine the search, and database-specific filters were applied where appropriate. Additionally, reference lists of included studies were manually screened to identify any further relevant articles. The complete search strategy, including exact search strings and filters for each database, is provided in the Appendices.

Study Selection Process

All identified records were imported into EndNote X9 (Clarivate, Philadelphia, PA) for reference management, and duplicate entries were systematically removed. The remaining studies were then screened in two stages. First, titles and abstracts were independently assessed to exclude irrelevant studies. Subsequently, full-text articles of potentially eligible studies were retrieved and evaluated against the predefined inclusion and exclusion criteria. Any discrepancies during the selection process were resolved through discussion to reach a consensus.

Data Extraction

Data extraction was performed independently by two reviewers (EEAE and MAMR) using a standardized and pre-piloted data extraction form to ensure consistency and completeness. Any discrepancies between the two reviewers were resolved through discussion, and when consensus could not be reached, a third reviewer (EBHB) was consulted to arbitrate. Inter-reviewer agreement prior to resolution was assessed using Cohen's kappa statistic (κ = 0.89), indicating excellent agreement. Extracted information included study characteristics (e.g., author, year, country, study design), population details, imaging modality, type of AI model, and diagnostic performance metrics such as sensitivity, specificity, accuracy, AUROC, and F1-score, where available. Additional data regarding comparisons with radiologists and validation methods were also collected to enable a comprehensive evaluation of model performance.

Quality Assessment and Risk of Bias

The methodological quality and risk of bias of the included studies were assessed using the QUADAS-2 (Quality Assessment of Diagnostic Accuracy Studies-2) tool [10]. This validated instrument evaluates four key domains: patient selection, index test, reference standard, and flow and timing. Each domain was assessed for risk of bias and applicability concerns. The use of QUADAS-2 ensured a systematic and transparent appraisal of study quality, facilitating the interpretation of findings in the context of potential methodological limitations.

Data Synthesis and Analysis

A qualitative synthesis of the included studies was conducted, focusing on the diagnostic performance of AI models for early lung cancer detection. Although a meta-analysis is often desirable in systematic reviews of diagnostic accuracy, it was not performed in this study due to substantial heterogeneity across the included studies. This heterogeneity arose from variations in AI model architectures, imaging modalities, dataset characteristics, threshold definitions, validation techniques, and reporting of performance metrics. Additionally, the inconsistent availability of key statistical parameters and the lack of standardized evaluation frameworks further limited the feasibility of pooling results quantitatively. Conducting a meta-analysis under such conditions could yield misleading or non-generalizable conclusions; therefore, a narrative synthesis was deemed more appropriate to provide a nuanced and accurate interpretation of the evidence.

Results

Study Selection Process

**Figure 1:**
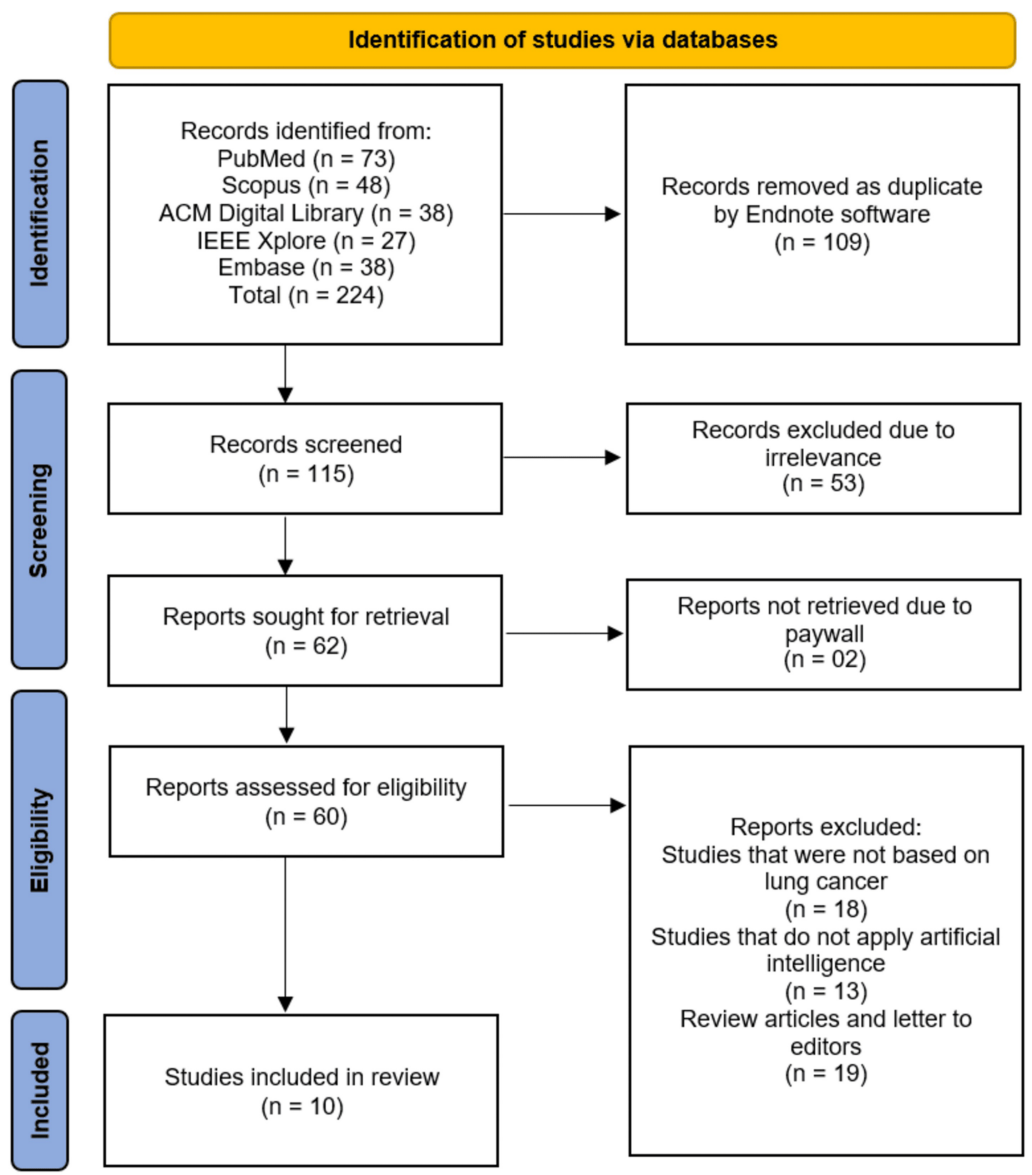
Study identification illustrated on PRISMA flowchart. PRISMA: Preferred Reporting Items for Systematic Reviews and Meta-Analyses;

A total of 224 records were initially identified through database searches, comprising 73 records from PubMed, 48 from Scopus, 38 from ACM Digital Library, 27 from IEEE Xplore, and 38 from Embase. After removing 109 duplicate records using EndNote software, 115 records remained for screening. Following title and abstract screening, 53 records were excluded due to irrelevance to the review question. The remaining 62 reports were sought for retrieval, of which two could not be accessed due to paywall restrictions. Subsequently, 60 reports were assessed for full-text eligibility. Of these, 18 were excluded because they were not based on lung cancer, 13 were excluded as they did not apply AI, and 19 were excluded as they were review articles or letters to the editor. Ultimately, 10 studies [11-20] met the predefined inclusion criteria and were included in this systematic review (Figure 1).

Study Characteristics and Design

A total of 10 studies [11-20], published between 2021 and 2025, were included in this systematic review. The characteristics of these studies are summarized in Table 2. The included studies were diverse in terms of design, population, imaging modality, and AI architecture, allowing for a comprehensive evaluation of the current state of AI in early lung cancer detection. The included studies employed a variety of designs, with the majority being retrospective analyses [11,13,15-20] and some described as experimental or prospective deep learning studies [12,14]. The sample sizes varied considerably, ranging from small-scale studies with 13 patients [13] to large, multi-cohort investigations involving over 9,000 participants [16]. The target populations were heterogeneous, encompassing mixed populations [11,12], individuals undergoing screening for lung cancer (including high-risk smokers) [14,16,18-20], patients with incidentally found lung nodules [13], and specific groups such as those with non-small cell lung cancer (NSCLC) [15]. CT, including LDCT, was the most frequently used imaging modality [11,12,14,16,18-20], followed by chest X-rays (CXR) [13,17]; one study additionally evaluated AI on programmed death-ligand 1 (PD-L1) immunohistochemistry whole-slide images, which fall under digital pathology rather than medical imaging [15].

**Table 2:** Characteristics of included studies. AI: artificial intelligence; CNN: convolutional neural network; VGG16: Visual Geometry Group 16-layer Network; GA-UNet3+: Genetic Algorithm-optimized U-Net 3+; ResNet34/50: Residual Network (34/50 layers); Lunit: Commercial AI software (Lunit INSIGHT); R-CNN: region-based convolutional neural network; Faster R-CNN: Faster region-based convolutional neural network; ESVM: ensemble support vector machine; MPSNLO: multi-phase suspicious nodule localization optimization; DL: deep learning; CT: computed tomography; LDCT: low-dose computed tomography; MDCT: multidetector computed tomography; CXR: chest X-ray; WSI: whole slide image; PD-L1: programmed death-ligand 1; IHC: immunohistochemistry; NSCLC: non-small cell lung cancer; U-Net: U-shaped convolutional neural network architecture; NLST: National Lung Screening Trial; DLCST: Danish Lung Cancer Screening Trial; LIDC-IDRI: Lung Image Database Consortium and Image Database Resource Initiative; LUNA16: Lung Nodule Analysis 2016 Challenge Dataset; NHS: National Health Service; CV: cross-validation; pts: patients; imgs: images.

Study (Author, Year)	Country	Study design	Sample size	Population	Imaging modality	AI model type	Dataset source	Validation method
Klangbunrueang et al. [11] (2025)	Thailand	Retrospective AI study	110/1097	Mixed	CT	CNN (VGG16)	Public	Internal
Said et al. [12] (2025)	Saudi Arabia	Experimental	1018 pts; 267 imgs	Mixed	CT	CNN (GA-UNet3+)	Public	Internal + External
Kwak et al. [13] (2023)	South Korea	Retrospective	13 patients	Incidental (non-screening)	CXR	CNN (ResNet34, Lunit v2–v3)	Single hospital (private)	Internal only
Sengodan et al. [14] (2023)	USA (LIDC-IDRI)	Experimental DL	1010 pts/1018 scans (888 used)	Screening lung nodules	CT	Hybrid (Faster R-CNN + ESVM + MPSNLO)	Public (LIDC-IDRI)	Internal (80:20 + CV)
Wu et al. [15] (2022)	China	Retrospective validation study	173 WSIs (train); 114–110–40–100 WSIs (tests)	NSCLC (resected + biopsy)	PD-L1 IHC whole-slide images (WSI)	U-Net CNN (DL segmentation)	Private hospital datasets	Internal + cross-assay (SP263) validation
Venkadesh et al. [16] (2021)	USA + Denmark	Retrospective (multicohort)	NLST: 9421; DLCST: 2052 (883 nodules)	Screening (high-risk)	LDCT	2D+3D CNN (ResNet50 + Inception-v1)	NLST (public) + DLCST	10-fold CV + external
Tam et al. [17] (2021)	UK	Retrospective	396 (198/198)	Lung cancer + matched controls	CXR	Ensemble CNN (Behold.ai)	Single-center NHS (private)	Internal validation
Chamberlin et al. [18] (2021)	USA (multi-country validation)	Retrospective cohort	117 (96 final)	LDCT screening (high-risk smokers)	LDCT	Hybrid CNN (ResNet + U-Net, ensemble)	Institutional + multicenter	Internal + external
Trajanovski et al. [19] (2021)	Multi-country (USA-based datasets)	Retrospective ML study	~8,600 CT scans	Screening/high-risk	CT	CNN + ResNet hybrid	Public + private	Internal + external (CV + independent datasets)
Gürsoy Çoruh et al. [20] (2021)	China	Retrospective	158 pts/158 nodules	High-risk/hospital	CT (MDCT)	CNN ensemble (Faster/Mask/Retina R-CNN)	Private + LUNA16	10-fold CV + benchmark

Convolutional neural networks (CNNs) were the dominant AI architecture, often in various hybrid or ensemble configurations. Several studies utilized established CNN architectures like VGG16 [11], ResNet [13,16,18], and U-Net [15,18]. Others proposed novel, more complex models, such as a GA-UNet3+ optimized by a genetic algorithm [12], a hybrid model combining Faster R-CNN with ensemble support vector machines (MPSNLO) [14], and an ensemble of 2D and 3D CNNs [16]. The sources of datasets were a mix of public repositories (e.g., Lung Image Database Consortium and Image Database Resource Initiative (LIDC-IDRI) and National Lung Screening Trial (NLST)) [11,12,14,16,19,20] and private institutional or single-center data [13,15,17,18]. Validation methods were robust, with most studies employing internal validation [11-20], and several also incorporating external validation using independent datasets to demonstrate generalizability [12,16,18,19].

Diagnostic Performance of AI Models

The diagnostic performance of AI models varied across studies, reflecting differences in task, model architecture, and clinical setting. Several studies reported high accuracy, sensitivity, and specificity. For instance, Klangbunrueang et al. [11] achieved a classification accuracy of 98.18% using a VGG16 model for CT scan image classification. Similarly, Said et al. [12] reported a high segmentation accuracy of 99.17% with their GA-UNet3+ model. Sengodan et al. [14] demonstrated exceptional performance for nodule detection and classification, with a sensitivity of 99.3%, specificity of 98.03%, accuracy of 98.53%, and an AUROC of 0.98. In the context of NSCLC, Wu et al. [15] used a U-Net model to assess PD-L1 expression on whole-slide images, achieving high concordance with pathologists and accuracy ranging from 93.3% to 96.2% (Table 3).

**Table 3:** Diagnostic performance of AI models for early lung cancer detection. CNN: convolutional neural network; VGG16: Visual Geometry Group 16-layer Network; GA-UNet3+: Genetic Algorithm-optimized U-Net 3+; ResNet34/50: Residual Network (34/50 layers); CXR: chest X-ray; MNPS-MEFL: Multi-Nodule Prediction System with Multi-Feature Learning; R-CNN: region-based convolutional neural network; Faster R-CNN: faster region-based convolutional neural network; DBN: deep belief network; ESVM: ensemble support vector machine; MPSNLO: multi-phase suspicious nodule localization optimization; DL: deep learning; U-Net: U-shaped convolutional neural network architecture; TPS: tumor proportion score; PD-L1: programmed death-ligand 1; WSI: whole slide image; ICC: intraclass correlation coefficient; R: correlation coefficient; 2D/3D: two-dimensional/three-dimensional; NCG: nodule candidate generation; FPR: false positive reduction; AI-RAD: artificial intelligence radiology model; MIL: multiple instance learning; SVM: support vector machine; GPS: Global Positioning System (feature fusion context); AUROC/AUC: area under the receiver operating characteristic curve; NR: not reported; sens: sensitivity; spec: specificity; acc: accuracy; rad: radiologist; κ: Kappa statistic; FP: false positive; LHMC: Lahey Hospital and Medical Center; UCM: University of Chicago.

Study (Author, Year)	Model architecture	Task	Sensitivity (%)	Specificity (%)	Accuracy (%)	AUROC	Comparison with radiologists	Key findings
Klangbunrueang et al. [11] (2025)	VGG16 (CNN)	Classification	NR	NR	98.18	NR	NR	Best model; high accuracy & stable
Said et al. [12] (2025)	GA-UNet3+	Segmentation	NR	NR	99.17	NR	NR	GA-optimized, smaller model, high precision, robust across datasets
Kwak et al. [13] (2023)	Lunit INSIGHT CXR (ResNet34-based)	Nodule detection	NR (13/13 detected)	NR	NR	NR	AI detected nodules earlier than the radiology report in 61.5% cases	Detected all incidentally found lung cancers; enabled earlier recognition, but no formal accuracy metrics reported
Sengodan et al. [14] (2023)	MNPS-MEFL (Faster R-CNN + DBN + ESVM + MPSNLO)	Detect + classify nodules	99.3	98.03	98.53	0.98	Not reported	Best performance; very high accuracy, strong ROC, low error, outperformed other AI models
Wu et al. [15] (2022)	U-Net (DL, residual)	Tumor seg + TPS (PD-L1 WSI)	86.1-88.8	95.9-97.9	93.3-96.2	NR	Vs. pathologists (R up to 0.9787; ICC 0.92-0.96)	High acc, strong path concordance, robust across 22C3/SP263, AI reduces time
Venkadesh et al. [16] (2021)	2D ResNet50 + 3D Inception-v1 (ensemble CNN)	Malignancy prediction (benign vs. malignant nodules)	84	90	NR	0.93	Comparable to radiologists; > PanCan model	Strong DL performance; generalizable; improved risk prediction over PanCan; radiologist-level accuracy
Tam et al. [17] (2021)	Ensemble deep CNN (Behold.ai Red Dot)	CXR lung cancer detection	80	93	87	NR	≈Rad accuracy; ↑ sens vs. 2/3 radiologists; ↓ spec slightly	AI = rad-level acc; best in AI+rad workflow; ↓ missed cancers; ↑ sens in triage use
Chamberlin et al. [18] (2021)	ResNet CNN + 3D NCG/FPR + encoder-decoder (AI-RAD)	Nodule detection	100	70.8	~88	0.942 (lung cancer pred.)	κ=0.741; mod agree	High sens, low FP spec; strong cancer prediction; screening tool
Trajanovski et al. [19] (2021)	Two-stage DL (detect_CNN / detect_SVM + ResNet-like MIL; ensemble best)	Scan-level detection	NR	NR	NR	0.86-0.94 (best ~0.938)	Comparable or better than radiologists (UCM 81 scans)	Strong generalization across NLST, LHMC, UCM; CNN detector > SVM; ensemble best; outperforms Kaggle model
Gürsoy Çoruh et al. [20] (2021)	CNN fusion (Retina, R-CNN, U-Net + GPS)	Malig. class (benign vs. malignant)	92.2	58.7	75.2	0.79	Rad AUC 0.87-0.92, Acc 86.9-89.9	AI = high sens, low spec; Rads better overall

Other studies focused on malignancy risk estimation and nodule detection. Venkadesh et al. [16] developed an ensemble CNN that achieved a sensitivity of 84%, specificity of 90%, and an AUROC of 0.93 for malignancy prediction, demonstrating performance comparable to that of radiologists. For lung cancer detection on CXRs, Tam et al. [17] reported that their ensemble deep CNN had a sensitivity of 80% and specificity of 93%, achieving accuracy similar to that of radiologists. Chamberlin et al. [18] reported a high sensitivity of 100% for nodule detection but a lower specificity of 70.8%, with an AUROC of 0.942 for lung cancer prediction. Trajanovski et al. [19] found that their two-stage deep learning model achieved scan-level AUROC values between 0.86 and 0.94, which was comparable to or better than radiologist performance. Conversely, Gürsoy Çoruh et al. [20] reported that their CNN fusion model had high sensitivity (92.2%) but lower specificity (58.7%), with an overall accuracy of 75.2% and an AUROC of 0.79, which was lower than the radiologists' performance in their study (area under the curve (AUC): 0.87-0.92).

Comparison With Radiologist Performance

A key objective of several studies was to compare AI performance against human radiologists. Venkadesh et al. [16] and Tam et al. [17] both found that their AI models achieved diagnostic accuracy comparable to that of radiologists. Tam et al. [17] further noted that an AI-assisted workflow improved sensitivity and reduced missed cancers. Kwak et al. [13] reported that their AI system detected nodules earlier than the original radiology reports in 61.5% of cases, highlighting AI's potential for earlier recognition of incidentally found lung cancers. Chamberlin et al. [18] found moderate agreement (κ = 0.741) between their AI model and radiologists, while Wu et al. [15] reported strong pathologist-AI concordance with intraclass correlation coefficients (ICC) ranging from 0.92 to 0.96 for PD-L1 scoring. In contrast, Gürsoy Çoruh et al. [20] found that while their AI model had higher sensitivity, radiologists demonstrated superior overall accuracy and specificity. These findings suggest that while AI can match or, in some cases, exceed radiologist performance for specific tasks, its role may be optimally realized as a complementary tool to enhance human interpretation.

Risk of Bias Assessment

Overall, the methodological quality of the included studies was moderate to high. For the patient selection domain, seven studies were rated as having low risk of bias [12,14,16,18-20], while Kwak et al. [13] was rated as high risk due to its highly selective, single-center sample of incidentally found lung cancers, and two studies were rated as unclear due to insufficient reporting of selection criteria [11,17]. All 10 studies demonstrated low risk of bias for the index test domain, indicating clear and reproducible descriptions of AI model architectures and outcomes [11-20]. For the reference standard domain, eight studies were rated as low risk [12-20], while two studies were rated as unclear due to incomplete specification of the reference standard against which AI outputs were compared [11,18]. All 10 studies demonstrated low risk of bias for the flow and timing domain, reflecting appropriate patient flow, consistent application of reference standards, and inclusion of all cases in analyses [11-20]. Regarding applicability concerns, most studies were rated as low across all three domains, indicating that the populations, index tests, and reference standards were well aligned with the review question. The only exception was Kwak et al. [13], which raised high applicability concerns for patient selection and unclear applicability concerns for the index test due to the highly selective population and the absence of formal diagnostic accuracy metrics (Table 4).

**Table 4:** Risk of bias and applicability concerns using QUADAS-2. QUADAS-2: Quality Assessment of Diagnostic Accuracy Studies-2.

Study (Author, Year)	Risk of bias				Applicability concerns		
	Patient selection	Index test	Reference standard	Flow and timing	Patient selection	Index test	Reference standard
Klangbunrueang et al. [11] (2025)	Unclear	Low	Unclear	Low	Low	Low	Low
Said et al. [12] (2025)	Low	Low	Low	Low	Low	Low	Low
Kwak et al. [13] (2023)	High	Low	Low	Low	High	Unclear	Low
Sengodan et al. [14] (2023)	Low	Low	Low	Low	Low	Low	Low
Wu et al. [15] (2022)	Unclear	Low	Low	Low	Low	Low	Low
Venkadesh et al. [16] (2021)	Low	Low	Low	Low	Low	Low	Low
Tam et al. [17] (2021)	Unclear	Low	Low	Low	Low	Low	Low
Chamberlin et al. [18] (2021)	Low	Low	Unclear	Low	Low	Low	Low
Trajanovski et al. [19] (2021)	Low	Low	Low	Low	Low	Low	Low
Gürsoy Çoruh et al. [20] (2021)	Low	Low	Low	Low	Low	Low	Low

Discussion

This systematic review synthesized evidence from 10 studies published between 2021 and 2025 to evaluate the accuracy of AI models for early lung cancer detection. The findings demonstrate that AI, particularly deep learning architectures such as CNNs, has achieved remarkable diagnostic performance across diverse clinical contexts. The reported accuracy metrics were consistently high, with several studies achieving accuracy exceeding 98% and AUROC values approaching or surpassing 0.94, underscoring the potential of AI to serve as a powerful adjunctive tool. However, the review also revealed considerable heterogeneity in study design, reference standards, and validation methodologies, highlighting both the promise and the current limitations of the evidence base.

AI Performance in Diagnostic Tasks

The performance of AI models in this review aligns with the broader trajectory observed in the field of medical imaging. The high accuracy reported by Klangbunrueang et al. [11] using a VGG16 architecture for CT scan classification (98.18%) and by Sengodan et al. [14] with their ensemble model combining Faster R-CNN and support vector machines (98.53%) reflects the capacity of deep learning to extract subtle imaging features that may elude conventional analysis. These findings are consistent with those of Ardila et al. [21], who demonstrated that a deep learning model could achieve an AUROC of 0.94 for lung cancer detection on LDCT scans. Similarly, the segmentation accuracy of 99.17% reported by Said et al. [12] using a genetic algorithm-optimized UNet3+ architecture mirrors the results of Hofmanninger et al. [22], who found that automated lung segmentation could achieve near-perfect Dice similarity coefficients across heterogeneous CT datasets.

One of the most striking findings is the high sensitivity achieved by several AI models, particularly for nodule detection and malignancy classification. Chamberlin et al. [18] reported a sensitivity of 100% for nodule detection on LDCT, albeit with a lower specificity of 70.8%, while Gürsoy Çoruh et al. [20] reported a sensitivity of 92.2% for malignancy classification. These results are consistent with Nam et al. [23], who evaluated a commercial AI algorithm for pulmonary nodule detection on chest radiographs (sensitivity: 91.9%, specificity: 80.6%). The high sensitivity is clinically significant, as early detection critically depends on minimizing false negatives. However, the accompanying trade-off with specificity raises considerations regarding overdiagnosis and unnecessary procedures, underscoring the need for implementation strategies that leverage AI as a triage tool rather than a standalone diagnostic device, a concept supported by Tam et al. [17], who demonstrated that AI-assisted workflows improved sensitivity and reduced missed cancers.

Comparison With Radiologist Performance

The comparison of AI performance with human radiologists yielded nuanced findings. Venkadesh et al. [16] and Tam et al. [17] both found that AI achieved diagnostic accuracy comparable to that of radiologists, while Kwak et al. [13] reported that AI detected incidentally found lung cancers earlier than radiology reports in 61.5% of cases. These findings align with McKinney et al. [24], who demonstrated that an AI system could outperform radiologists in breast cancer detection. Conversely, Gürsoy Çoruh et al. [20] found that while their AI model had higher sensitivity, radiologists demonstrated superior overall accuracy and specificity. This discrepancy may reflect differences in task complexity, reference standard quality, or AI architecture. Importantly, AI and radiologists may possess complementary strengths: AI excels at pattern recognition and sensitivity, while radiologists contribute contextual knowledge, clinical correlation, and higher specificity. This complementarity is consistent with Sim et al. [25], who reported that AI-augmented workflows improved diagnostic accuracy and efficiency compared to either alone.

Model Architecture Trends

The diversity of AI architectures represented in this review reflects the rapid evolution of the field. While CNNs remain foundational, ensemble methods and hybrid approaches have enabled incremental performance gains. The ensemble model developed by Trajanovski et al. [19], which combined CNN-based detection with ResNet-like multiple instance learning, achieved scan-level AUROC values up to 0.938, outperforming individual models and even a Kaggle competition-winning approach. Similarly, the hybrid model of Sengodan et al. [14] combined Faster R-CNN with ensemble learning to achieve state-of-the-art performance. These findings resonate with Causey et al. [26], who demonstrated that ensemble deep learning models for lung nodule classification outperformed individual architectures. The consistent finding that ensemble and hybrid models yield superior performance suggests that future AI development should prioritize model integration.

Generalizability and External Validation

The generalizability of AI models is critically dependent on validation methodology. Several studies employed external validation using independent datasets, a practice essential for assessing real-world performance. Venkadesh et al. [16] validated their model on both the NLST and Danish Lung Cancer Screening Trial (DLCST) cohorts, demonstrating robustness across different screening populations. Trajanovski et al. [19] similarly validated their model across multiple datasets, including NLST, Lahey Hospital and Medical Center (LHMC), and University of Chicago (UCM), and found consistent performance. The importance of external validation is underscored by Zech et al. [27], who demonstrated that deep learning models often exhibit significant performance degradation when applied to datasets from different institutions. The inclusion of external validation in several studies strengthens the evidence base, yet the fact that not all studies incorporated this step highlights an area for improvement.

Methodological Quality and Risk of Bias

The QUADAS-2 risk of bias assessment revealed several methodological considerations. Patient selection was the domain most frequently rated as having unclear or high risk of bias, primarily due to retrospective designs and selective sampling. Kwak et al. [13], for example, used a highly selective sample of incidentally found resectable lung cancers from a single center, limiting generalizability. This finding is consistent with Liu et al. [28], who found that most deep learning studies in medical imaging were retrospective and at high risk of bias. The reference standard domain also presented concerns in two studies [11,18], where the ground truth was incompletely specified, a problem that can introduce verification bias. Future studies should adhere to reporting guidelines such as STARD (Standards for Reporting of Diagnostic Accuracy Studies) and CLAIM (Checklist for Artificial Intelligence in Medical Imaging) to enhance transparency.

Heterogeneity in outcome reporting is another notable finding. While some studies reported comprehensive metrics, including sensitivity, specificity, and AUROC, others, such as Kwak et al. [13], reported only descriptive findings without formal accuracy metrics. This variability limits quantitative synthesis and meta-analysis, a challenge recognized in the field. The SPIRIT-AI and CONSORT-AI initiatives aim to standardize reporting of AI interventions, and their adoption would facilitate evidence synthesis and robust comparisons.

Clinical Implications

The findings support the integration of AI into early lung cancer detection pathways, but with important caveats. The high sensitivity of many AI models suggests they could serve as triage tools to prioritize suspicious cases for radiologist review, a workflow evaluated by Tam et al. [17] and found to improve overall diagnostic performance. Additionally, the capacity of AI to detect nodules earlier than routine radiology reporting, as demonstrated by Kwak et al. [13], suggests potential for reducing diagnostic delays, particularly in high-workload settings. However, the sensitivity-specificity trade-off underscores the importance of human oversight to mitigate false positives. The optimal integration of AI may therefore be as a second reader or decision support tool rather than a replacement for radiologist expertise, a conclusion aligning with the American College of Radiology position statements.

Beyond Radiology: Digital Pathology Applications

The inclusion of Wu et al. [15] broadens the scope beyond traditional imaging to digital pathology for PD-L1 expression assessment in NSCLC. This study achieved high concordance with pathologists (ICC: 0.92-0.96) and accuracy ranging from 93.3% to 96.2%, demonstrating that AI can reliably quantify immunohistochemical markers critical for treatment selection. These findings are consistent with Coudray et al. [29], who demonstrated that deep learning could classify lung cancer histology and predict genetic mutations from histopathology images. The extension of AI from detection to biomarker quantification represents a promising frontier that could enhance precision oncology through more objective, reproducible, and faster assessments.

Limitations

This systematic review has several limitations that should be considered when interpreting the findings. First, the review was limited to five databases (PubMed, Scopus, ACM Digital Library, IEEE Xplore, and Embase), and while this captures a broad range of biomedical and technical literature, relevant studies from other sources, such as grey literature or conference proceedings, may have been omitted. Second, substantial heterogeneity in study design, imaging modalities, AI architectures, outcome measures, and reference standards precluded quantitative meta-analysis, limiting the ability to generate pooled estimates of diagnostic accuracy. Third, the majority of included studies were retrospective in design, which may introduce selection bias and limit the generalizability of findings to prospective clinical settings. Fourth, some studies lacked external validation, raising questions about the robustness and generalizability of their reported performance. Fifth, the risk of bias assessment identified concerns in patient selection and reference standard domains for several studies, indicating that methodological quality varied across the evidence base. Sixth, publication bias remains a concern, as studies with favorable results are more likely to be published, potentially leading to overestimation of AI performance. Finally, the included studies focused predominantly on AI for detection and classification tasks, with limited evidence on the integration of AI into clinical workflows or its impact on patient outcomes such as mortality or quality of life.

## Conclusions

AI, particularly deep learning models such as CNNs and their hybrid variants, holds strong potential for supporting early lung cancer detection across different imaging modalities and clinical settings. Current evidence suggests that these tools are most appropriately positioned as complementary systems to assist clinicians by enhancing diagnostic workflows and supporting decision-making rather than serving as standalone diagnostic replacements. However, the existing literature is marked by notable heterogeneity in study designs, validation approaches, and reporting practices, along with methodological limitations that may affect the robustness and generalizability of findings. Therefore, future research should focus on prospective study designs, standardized reporting in line with established guidelines, and rigorous external validation across diverse populations to strengthen clinical applicability. In addition, further work is needed to assess the real-world clinical impact of AI systems, including their effect on diagnostic pathways and patient outcomes, while also expanding applications toward biomarker quantification and multimodal data integration to advance precision lung cancer care.

## Appendices

**Table 5:** Detailed search strings and filters used for each database.

Database	Search string	Filters applied	Results
PubMed	(("lung cancer"[MeSH Terms] OR "lung neoplasms"[MeSH Terms] OR "lung cancer"[Title/Abstract] OR "pulmonary nodule"[Title/Abstract]) AND ("artificial intelligence"[MeSH Terms] OR "deep learning"[MeSH Terms] OR "machine learning"[MeSH Terms] OR "convolutional neural network"[Title/Abstract] OR "CNN"[Title/Abstract] OR "AI"[Title/Abstract]) AND ("early detection"[MeSH Terms] OR "early diagnosis"[MeSH Terms] OR "screening"[Title/Abstract] OR "early detection"[Title/Abstract]))	English, 2021/01/01 - 2025/12/31	73
Scopus	( TITLE-ABS-KEY("lung cancer" OR "lung neoplasm" OR "pulmonary nodule") AND TITLE-ABS-KEY("artificial intelligence" OR "deep learning" OR "machine learning" OR "convolutional neural network" OR "CNN" OR "AI") AND TITLE-ABS-KEY("early detection" OR "early diagnosis" OR "screening") )	English, 2021 - 2025	48
Embase	('lung cancer'/exp OR 'lung neoplasm'/exp OR 'lung cancer':ab,ti OR 'pulmonary nodule':ab,ti) AND ('artificial intelligence'/exp OR 'deep learning'/exp OR 'machine learning'/exp OR 'convolutional neural network':ab,ti OR 'cnn':ab,ti OR 'ai':ab,ti) AND ('early detection'/exp OR 'early diagnosis'/exp OR 'screening':ab,ti)	English, 2021 - 2025	38
IEEE Xplore	("lung cancer" OR "lung neoplasm" OR "pulmonary nodule") AND ("artificial intelligence" OR "deep learning" OR "machine learning" OR "convolutional neural network" OR "CNN" OR "AI") AND ("early detection" OR "early diagnosis" OR "screening")	English, 2021 - 2025	27
ACM Digital Library	Abstract:("lung cancer" OR "pulmonary nodule") AND Abstract:("deep learning" OR "CNN" OR "artificial intelligence") AND Abstract:("early detection" OR "screening")	English, 2021 - 2025	38
